# Bibliographical Notices

**Published:** 1842-05-21

**Authors:** 


### BIBLIOGRAPHICAL NOTICES.

1. *An Exposition of the Unjust and Injurious Relations of the U. S. Medical Corps. By a Member.* Baltimore, 1842.
2. *Remarks on the Pay of Surgeons of the Navy.* (A schedule of an intended petition to Congress.)
3. *Information for Persons desirous of entering the Medical Department of the Navy.* (Circular of the Navy Department.)

The modest, gentlemanly, and exceedingly well written little pamphlet of twenty-two pages, which stands at the head of our list of promptings to this article, richly deserves to be transferred entire to the pages of every medical journal in the country; and it is with extreme regret that we feel compelled by our narrow limits to deny it the means of more extended circulation in the Examiner. Some extracts, and an analysis of its most important contents are all that we can offer; but we must be permitted to express, in the very preface of our remarks, the gratification derived from perusing the short but generous and expostulatory notice which it has received from the New York Medical Gazette, (No. 17,) and the surprise experienced in observing the manner in which our usually amiable friend and cotemporary of the Boston Journal (No. 13) has seen fit to treat an exposé of abuses most seriously effecting the interests of not only a large and most respectable body of professional men, but also, the nation itself, through the right arm of its defence. If a case of absurd neglect of human life and safety was ever clearly established it is so by the facts of our author, proving what we well knew before—though a mere civilian, and ignorant of ships of war except as an "idler,"—the total want of a fixed position, *enjoyed by right*, for the American naval surgeon at sea. So strikingly were these abuses forced upon our attention during a very short practical observation of naval discipline, that we had formed the intention of commenting upon them, long before the appearance of the essay of "a Member;" but have been deterred by the hopelessness of any reform under the imbecile management to which, until recently, our glorious but unfortunate little navy has groaned for years. The pamphlet under notice commences with the following paragraph:

"Since the entrance of the present head of the Navy Department upon the duties of his office, he has manifested such a knowledge of the various interests and necessities of the service committed to his charge, and such an energetic spirit of improvement and reformation, that to address him with a view to imparting information, or stimulating his action, may have the appearance of presumption; but his course has so far inspirited the almost wasted hopes of the various branches of the service, that those who heretofore have shut their eyes to error, from despair of its correction, now feel encouraged to point it out, and they confidently rely upon the courtesy which marks his official intercourse with the members of the service, to view their intentions graciously, although they may be superfluous."

This compliment to the present incumbent we hope and believe to be just; nor would it have been inappropriately addressed to his immediate predecessor, whose short lived tenure of office sufficed only to awaken fond anticipations which he was not permitted to gratify. It is not unreasonable, then, to hope that some practical benefit may result to the service from the public agitation of the subject at the present time. But the editor of the Boston Journal objects to the anonymous character of the publication of "A Member!" His official station brings him closely into contact with seamen in the merchant service, and he can hardly be entirely ignorant of the necessary despotism of maritime life, which is carried still further in some respects in the navy. Does he not know that every officer is bound by an imperative law to publish no ill of government or of the department to which he is subservient? When a subordinate dares to point out the errors of his superior before a popular tribunal, it behoves him to guard his position with all possible precaution. Even in communicating directly with the head of the Department, as it is his duty to do, his safety from serious consequences often depends on the character of those who happen to be " dressed in a little brief authority" for the time being; and it may be, sometimes, that the greater the truth of a complaint the greater is the libel esteemed.* We do not suppose that any such motive has induced the suppression of the name of the able and gentlemanly writer under notice;—modesty is a more probable motive—but even supposing it otherwise, the anonymous character of the pamphlet abstracts nothing from its authority, and should be regarded only as a proof of reasonable discretion. Be this as it may, *we* are certainly under no bond of law, honour, or courtesy, to avoid speaking of these things without reserve, and can fully endorse all the important statements and inferences of "A Member" from sufficient knowledge.

The difficulty of procuring a sufficient supply of assistant surgeons in the navy has been severely felt of late; and articles have appeared in the daily papers designed to entice new applicants. The unsuccessful result of such attempts has excited some surprise; but it is the natural consequence of the system against which the remarks of " A Member" are directed. About three-fourths of the applicants are rejected by the board of Examiners at the preliminary examination.

This board enjoys the well deserved reputation of being quite as independent of the extraneous influences of wealth, political power, party feeling, and personal friendships as any other bureau or sub-department of government. Its action is severe but just: it is designed to secure the services of highly competent and marked professional men: nor can this rigor be materially relaxed without fatal consequences; for, not only is the competition between rival medical schools daily vitiating the quality of medical instruction generally, as professional teaching gradually sinks into a trade, but the

* Communications from subordinates must, by the regulations of the navy, pass through the hands of the commanding officer of the ship or station, and are of course open to his censorship.

disadvantages of the position of the selected candidates is such, for many years after the appointment, that much higher and more thorough knowledge is required in the first instance to compensate for the loss of half the valuable time of the first ten years of professional life, devoted, by those on land, to studies rendered impossible to the assistant surgeon on ship-board.

The choice of men of superior abilities is therefore indispensable to the proper organization of the naval medical corps—men who are always in demand by land, and who can generally achieve ample incomes if led by their taste and wishes in that direction. Let us examine what temptations the naval service holds out to such men. Let us hear " A Member."

" It is evident that the government expects its youngest medical officer to be fully qualified as a physician and surgeon; to have his mind maturely formed and well cultivated; being properly satisfied of this, the legislature and executive of his country fulfil the promise made to his hopes, for although they do not give him the pay of a respectable clerkship, the President and Senate confer upon him an honorable *commission*, consistent with the character they have required him to meet. But here all justice stops; he has a commission in his pocket, it is true, but none in fact; caprice and usage are more powerful than his country's will. His commission is never known again while he is an assistant surgeon, perhaps, for ten or twelve long years of his life. On ship-board the privileges of that commission are violated; he is at once, suddenly, thrust from the station to which his profession, his age, the interests of the service, and the intentions of the government entitle him. Simple warrants given by the department, without the concurrence of the senate, take precedence of him; aye! even temporary *appointments*, made or destroyed by the breath of the ship's captain, are, in all the usages of sea life, superior to the rank of his commission.

"Upon reporting himself on ship-board, the assistant surgeon is assigned to the narrow limits of the steerage, crowded with boys about entering upon their nautical education; full of fun and frolic, and merry with the noisy hilarity of youth. This is his home, and these are his associates. If he is admitted into the society of those more nearly his equals in years, it is as a concession to which his rank, implied from his *apartment*, does not entitle him. Upon descending to this apartment, perhaps, the first salutation that he meets, is a laugh at his trunk of books. Where is he to put them? No trunks are allowed there, and the only accommodation that he has, is a "stow-hole" for his clothes. But it would be idle to make any provision for professional studies in a place so little suited to their prosecution. The student who has for years taught his mind to seek pleasure in the pursuit of knowledge, and strengthened it by converse with those of the highest order; regarding every day as lost in which he does not add to his own ability to be useful, finds himself compelled to recede from the station he had acquired, to return to his forgotten juvenility, and either, to repress all social feeling, or, to find its enjoyment by becoming again a boy. He is also compelled to submit to all the minute regulations established to restrain his more youthful associates. Human nature finds it easier to yield to surrounding circumstances, than, continually, to resist them, and the foregoing relations are sufficient to diminish the tone of thought and feeling of any mind. But the medical officer is expected to make bricks out of straw. Notwithstanding the adverse circumstances in which he is placed, and that such usages debar him of the means of improvement, he is expected, daily, to increase in knowledge, and at the end of five years, is submitted to another rigid examination to test his progress, and should he be again successful, is rewarded by a very slight increase of pay; but his station is in no wise changed. His apartment and his associations are still the same, save, that a new and more juvenile set may take the place of many of his former companions, who have now grown past him, and are occupying separate apartments in the ward-room, with all the concomitant honors of ward room officers; some of them, as has been the case, may be acting commanders, while yet he is the humble steerage officer. During the years that he passes in the rank of an assistant surgeon, heretofore, and most likely hereafter, seldom less than ten, there are constantly presented to him the following contrasts with his own situation.

"The commander of a squadron has a young friend who, before settling in life, wishes to see a little of the world; the commodore of his own free will makes him his secretary for the cruise, and he passes at once from civil life to the occupation and comforts of his own state-room, and to the honors and associations of the ward-room. All the other appointments from civil life, and which are made without any test of qualification, are also admitted at once to these superior honors and comforts; the professor of mathematics from his college or school room, the chaplain from his desk, the purser from his counting room, and a second lieutenant of marines from any occupation in life, from the mechanic's bench to the military academy. We do not adduce these as any instances of impropriety, so far as the officers named are concerned, but only to show the great wrong done the assistant surgeon, who enters the service as a commissioned officer, to become permanently identified with it; with a professional education, tested by rigid scrutiny, and who on the day that each of the above named officers is first admitted, may have been devoting his time and his talents to the service for five, eight or ten years."

Well may the author remark:—

"But the history of the success of the medical corps is not its whole history; there is a counter narration, unrecorded and undivulged. Who takes note of those whose talents would have been an honour and benefit to the country's service, but who, from familiarity with its injustice, shrink from its employ? We can adduce at least one eminent professor who carefully guards his students from the navy, as the mælstrom of talents, fame and fortune."

And it is the mælstrom of talent! Figure to yourself the condition of a past assistant surgeon, who has entered the medical corps at the age of twenty-three years, and has served for nearly or quite ten years. The wild young lads of sixteen or eighteen with whom he was formerly compelled to *play* at twenty-three, are now Lieutenants, and eagerly expecting command, with which they are not unfrequently charged as acting commanders; but another race of roystering boys are the mess-room mates of this man of middle life, and frolic with the gray hairs which already begin to variegate his ear-locks. Old as he is, he must play with them, or they will play with him. A dark and often damp apartment of the narrowest dimensions,—the home of songs and comic pranks,—is the common *study* of the middies and the inferior grade of medical officers. No state rooms accommodate the libraries of these gentlemen; and if they wish to carry with them some slender intellectual food, happy is he who finds room in his stow hole for a shelf to accommodate a dozen volumes, and in his birth for a few more, where he may repose uneasily upon his literary treasure or bruise his face against it in rough weather. Little other use can he make of it; for, in such a den, all study is out of the question.

And what advantages in money or fame are offered by his country to the medical aspirant in return for such an undignified and self-sacrificing position? The following is a fair picture of the share of naval honour which falls to the lot of the assistant surgeon.

"Not only do the superior advantages awarded these commissioned, warranted, and temporarily appointed officers over the assistant surgeons, give them greater comforts, but they are by consequence entitled to certain little ceremonies of honour which, shadowy and unsubstantial though they be, by being withheld from the medical officer, serve to mark the humility of his position. When named, these ceremonies become triflingly ridiculous, and to estimate them with becoming gravity it is necessary that they make part and parcel of an isolated society, made up of ceremonies as appeals to those who are accustomed to regard symbols as substance; and when such trifles confer honour, they imply corresponding degradation when they are withheld. The following are some of the observances alluded to: when a *ward-room* officer passes out of, or into the ship, a boatswain's mate attends the side and chirps him a note of honour; he approaches the ship on the starboard side, that sacred to rank; descends to his apartments by the *after*, instead of the *forward* hatchway; has the privilege of answering a proud "aye, aye" to the sentry's hail, and is lighted over the gangway by *two* lanterns. On the contrary, the assistant-surgeon, after years of service, passes out of or into the ship in silence and unhonoured, *must* approach it on the larboard side, or be regarded as a trespasser, and, to the sentry's hail, answer an humble "no, no;" must puff his cigar on the larboard side in company with his friends the middies, some of whom are now but little older than his own children; the sacred starboard being tabooed to his humility; and he must be careful to select the hatchway in descending to his apartment. As these rituals and petty distinctions are in incessant action, and are insisted upon, the whole life on shipboard is one of continued insult to the medical officer."

This picture of the honours attached to the service is certainly not very enticing, and were it as familiar to the ambitious young men of the profession as it should be, the acknowledged difficulty of procuring recruits for the corps would be greatly increased. It presents a view of that half of an adult apprenticeship which is usually passed at sea; but even on land, the naval assistant surgeon labours under difficulties fearful in comparison with those which surround the civil practitioner of the same age. Often attached to vessels or squadrons in port, to marine asylums, navy yards, or receiving ships, and liable at all times to a sudden change of residence under the order of the Department, he is usually located at a distance from the great libraries and hospitals, so readily accessible to others, and finds his time—except when waiting orders, or on long leave of absence—so far controlled and cut to pieces by the necessary restraints of discipline, that it is rendered in great degree useless for scientific improvement. Though some members of the corps have conquered those difficulties, and, under favourable circumstances, have risen in after life to wealth and high professional standing on shore in spite of them, those exceptions serve but to prove the rule that service in the navy is among the most unfortunate of medical positions.

That the army service is in any very great degree more desirable we are not prepared to say, and shall avoid all invidious comparisons between the usefulness and pleasure of these two modes of life.

But surely there must be something in the future more enticing, or why should any one be willing to encounter the mortifications which lie in the way of a member of the medical corps. Hear our author again:—

"At length, after years of habituation to such indignities, so as almost to lose sensibility to them, however keenly he may have felt them at first, the assistant is promoted to the rank of full surgeon, and enters the ward-room. It is well for his feelings that the education of humility he has passed through has prepared him for a patient endurance of its continuance.

The surgeon has now become a ward-room officer, and, excepting a trifling increase of his pay of two hundred dollars for every five years of service, his position is stationary; and stationary at the most humble point; the respect he receives is dependent entirely upon the character or the whim of his associates: none is guaranteed him by regulation, and he is generally compelled to yield precedence in all points of etiquette to the youngest of his associates, and even has his seat at the mess table assigned him upon this estimate of his rank. Gradually, and by the lapse of time, his brethren, the "sea officers," pass from the ward-room to the rank and honours of commanders, and to the occupation of their own cabins, but the old surgeon, though gray hairs and an enfeebled body mark the length and fidelity of his service, finds no increase of comfort; the same position and the same limited apartment which he entered on the first day of his promotion, are all that he can claim on the last day of his service, and he passes to his grave unhonoured, save by the spontaneous award of those who knew him. Such treatment and such relations are assigned to the medical officers of no other service in the world. In our own army the medical officers have a rank and comforts allotted them fairly proportioned to the respectability of their profession and the importance of their duties, and due to the self-respect which it should be the principle of the government to encourage in every officer; and among their brother officers of the army, they hold those social relations which cultivated minds always acknowledge as the claim of their education and profession. Surgeons in the army are assimilated to the rank of major and have choice of quarters after the commanding officer of the post."

It appears, then, that an anomalous position, with the allowance of a state room and the permission to take a station at the foot of the ward-room table—privileges of courtesy and not of right—must bound the social ambition of the naval surgeon. Truly it must require all the romance and love of travel peculiar to early life to tempt men of education and talents into this unpromising career; for, chance of high social rank, ample emolument, or extended fame, it offers none. Even romance and the love of travel are destined to bitter mortification: there is nothing romantic in the formal stiffness of etiquette indispensable in a vessel of war, and, when abroad, the responsibilities of the assistant surgeon are too numerous and pressing ever to permit him to wander far from his station while on duty. Many are led away by false ideas on this subject, and tempted at least to try the experiment: but, once in service, there is seldom a return—such are the habits of naval life, that a few years spent at sea for ever incapacitates most men for a successful career on land.

It would be contrary to all our feelings to endeavor to discourage our young medical men of talent from a pursuing naval life; and, were the evils that have been pointed out inevitable, the documents under notice would have escaped all comment from us: but it is clearly shown, by "A Member," that the unfortunate position of the medical corps is not the result of necessity, but of the absence of established regulations upon this subject. It is shown to be inconsistent with the wishes and intentions of Government—it is entirely corrected in Great Britain and some other maratime powers—and we may add, on our own account as a civilian, what it would be a breach of discipline in "A Member" to say, that the inexcusable neglect with which it has been heretofore treated in this country furnishes one of the strongest proofs of the proverbial weakness of those who have often been called to preside over the destinies of our noble but sadly mismanaged little navy. "A Member" seems to anticipate a brighter day.—We ardently hope and pray that he may not be disappointed. At present there is a vagueness in the limit of responsibility between the surgeon and the commander, which, coupled with the obvious injustice of permitting medical officers to be tried by court-martial in which there is no medical representation, subjects the sick or the criminal to the danger of tyrannical oppression when power happens to be vested in unworthy hands, and must sometimes lay the surgeon open to insult or loss of station for enacting fearlessly and conscienciously the duty of the man and the officer. If our Government wishes to secure sufficient, permanent and valuable accessions to the naval medical corps, these errors of regulation must be corrected; for, men of talents and dignity will not crowd forward to a station in which they find themselves underestimated in the social scale, and unprotected in their nicer feelings.

"A member" says something on the subject of pay—treating it very properly as a question altogether secondary in importance to those already mentioned. But its importance is not slight. We have nothing in common with those who talk largely of the liberality of the liberal professions while closely regarding their own interests,—lawyers legislating for physicians — politicians for the military — divines for the philanthropic, &c. There is no axiom more correct than that nothing long continues to be well done that is not well paid for, and the services of the medical corps are not well paid.

But we are warned by our familiar that our alloted space in this number is already filled; and, should we enter upon this delicate question at all, it must be in a future number. R. C.

